# Precise Species Identification by Whole-Genome Sequencing of *Enterobacter* Bloodstream Infection, China

**DOI:** 10.3201/eid2701.190154

**Published:** 2021-01

**Authors:** Wenjing Wu, Li Wei, Yu Feng, Yi Xie, Zhiyong Zong

**Affiliations:** West China Hospital, Sichuan University, Chengdu, China (W. Wu, L. Wei, Y. Feng, Y. Zie, Z. Zong);; State Key Laboratory of Biotherapy, Chengdu (W. Wu, Y. Feng, Z. Zong)

**Keywords:** Enterobacter, Enterobacter cloacae, bloodstream infection, bacteremia, bacteria, bacterial infections, antimicrobial resistance, China

## Abstract

The clinical importance of *Enterobacter* spp. remains unclear because phenotype-based *Enterobacter* species identification is unreliable. We performed a genomic study on 48 cases of *Enterobacter*-caused bloodstream infection by using in silico DNA–DNA hybridization to identify precise species. Strains belonged to 12 species; *Enterobacter xiangfangensis* (n = 21) and an unnamed species (taxon 1, n = 8) were dominant. Most (63.5%) *Enterobacter* strains (n = 349) with genomes in GenBank from human blood are *E. xiangfangensis*; taxon 1 (19.8%) was next most common. *E. xiangfangensis* and taxon 1 were associated with increased deaths (20.7% vs. 15.8%), lengthier hospitalizations (median 31 d vs. 19.5 d), and higher resistance to aztreonam, cefepime, ceftriaxone, piperacillin-tazobactam, and tobramycin. Strains belonged to 37 sequence types (STs); ST171 (*E. xiangfangensis*) was most common (n = 6). Four ST171 strains belonged to a defined clone. Precise species identification has greater implications for epidemiology and infection control than treatment.

*Enterobacter* spp. belongs to the family *Enterobacteriaceae* and is a common pathogen in a variety of infections, such as bloodstream and intraabdominal infections, most of which are healthcare associated (*1*). *Enterobacter* spp. is the third most common human pathogen, after *Escherichia coli* and *Klebsiella pneumoniae*, and is therefore of clinical importance (*1*). *Enterobacter* consists of several closely related species (*1*) that cannot typically be identified precisely by common phenotypic tests. The taxonomy of *Enterobacter* is complicated by the reassignment to other genera of some species that formerly belonged to the *Enterobacter* genus. For example, *E. aerogenes* has been moved to genus *Klebsiella* (*2*), *E. agglomerans* to genus *Pantoea* (*3*), and *E. sakazakii* to genus *Cronobacter* (*4*). Currently, 14 *Enterobacter* spp. with validly published names exist, and 3 additional *Enterobacter* spp. have tentative species designations awaiting validation under the rules of the International Code of Nomenclature of Bacteria (Bacteriological Code) (Appendix 1 Table 1).

Several *Enterobacter* spp., such as *E. asburiae, E. cloacae,* and *E. hormaechei*, cause infections in humans (*1*). *Enterobacter* strains extracted from clinical samples are usually reported as *E. cloacae,* and sometimes *E. asburiae*, *E. hormaechei,* or *E. kobei,* by automated microbial identification systems such as Vitek II (bioMérieux, https://www.biomerieux.com). However, such phenotype-based tests are unreliable for species identification of *Enterobacter* and can result in misidentification (*1*). For instance, all *Enterobacter* spp. have a positive reaction for β-galactosidase, arginine dihydrolase, citrate utilization, sucrose, amygdalin, arabinose, and D-glucose but are negative for lysine decarboxylase, H_2_S production, urease activity, indole production, deaminase, and gelatinase (*5*–*7*). Differentiating *Enterobacter* spp. by biochemical reactions commonly used in clinical microbiology laboratories is therefore difficult. The differences in clinical importance of each *Enterobacter* species remain largely unknown because they are regularly misidentified in clinical microbiology laboratories.

Because of the substantial reduction in cost of whole-genome sequencing for bacterial strains, we are entering the era of genomic microbiology (*8*). Newly created methods can determine the overall nucleotide identities between genome sequences and therefore enable more precise species identification (*9*). Calculation of average nucleotide identity (ANI) between genomes is widely used for species identification. It has been proposed that ANI >96% would guarantee species assignation, whereas ANI of <93% can be considered species differentiation (*10*). However, ANI values in the range of 93%–96% represent a vague zone in which the boundary of a species might fall (*10*). DNA–DNA hybridization (DDH) remains the standard for species identification, with a >70% cutoff recommended to define a species. However, DDH is cumbersome, prone to fluctuation, and requires the availability of type strains. To overcome the shortcomings of DDH, in silico DDH (isDDH) mimics DDH by comparing genome sequences and can be a reliable and convenient tool for species assignation. To provide insight into the potential clinical importance of different *Enterobacter* spp., we performed a genomic study using isDDH to identify bloodstream infection (BSI)–causing *Enterobacter* strains to the species level.

## Materials and Methods

### Strain and Susceptibility Tests

We collected nonduplicate *Enterobacter* strains recovered from blood cultures during January 2016–June 2018 at West China Hospital of Sichuan University (Appendix 2 Table 1). West China Hospital is a 5,000-bed major referral hospital in western China. Initial species identification and in vitro susceptibility testing were performed by using Vitek II. We determined MICs of colistin by using the broth microdilution method of the Clinical and Laboratory Standards Institute (CLSI) and interpreted susceptibility following CLSI guidelines (*11*). For colistin and tigecycline, no CLSI breakpoints are available, so we used breakpoint standards defined by the European Committee on Antimicrobial Susceptibility Testing (https://www.eucast.org). Multidrug resistance was defined based on the criteria for *Enterobacteriaceae* (*12*).

### Patient Data

West China Hospital has a comprehensive hospital information system, which allowed us to retrieve patient data including age, sex, length of hospitalization, and clinical outcomes (death or discharge) from electronic medical records. One patient (with strain 090040) had an unusually long hospital stay (578 d) because of a medical dispute and was removed from our analysis of length of stay. According to social customs in China, dying at home is preferred over the hospital; it is likely many patients chose to stop treatment and return home if treatment was not working and patients felt death was imminent. We categorized patients who chose to be discharged but were likely to die in the hospital (judged by the consensus of 2 physicians reviewing blind data) as patients with predicted death. BSI, the type of BSI (primary or secondary to infection of other sites), central line–associated BSI (CLABSI), and healthcare-associated infection were determined by using criteria established by the Centers for Disease Control and Prevention’s National Healthcare Safety Network (*13**,**14*). We conducted the study in accordance with the amended Declaration of Helsinki. The Ethics Committee of West China Hospital approved the study and waived informed consent.

### Short-Read Genome Sequencing, Analysis, and Precise Species Identification

All strains underwent whole-genome sequencing by using the HiSeq X10 platform (Illumina, https://www.illumina.com). Genomic DNA was prepared by using the QIAamp DNA mini kit (QIAGEN, https://www.qiagen.com). We used Unicycler version 0.4.3 (*15*), in the conservative mode for increased accuracy, to perform a de novo hybrid assembly. Precise species identification was established by determining the pairwise isDDH between the genome sequence of the query strain and those of type strains of *Enterobacter* spp., including the validly published species and the species awaiting validation (Appendix 1 Table 1). This process was performed by using the Genome-to-Genome Distance Calculator, formula 2 (*16*). A >70% cutoff was applied to define a species. In addition, we determined the pairwise ANI of the genome sequence of the query strain and those of type strains of *Enterobacter* spp. (Appendix 1 Table 1) by using JSpecies software (https://imedea.uib-csic.es/jspecies) with a >96% ANI cutoff to define a species (*10*). Sequence types (STs) were determined by using the genomic sequences to query the multilocus sequence typing database of *E. cloacae* (https://pubmlst.org/ecloacae). Antimicrobial resistance genes were identified from genome sequences by using the ABRicate program (https://github.com/tseemann/abricate) to query the ResFinder database (https://genomicepidemiology.org).

The genome sequences of all *Enterobacter* strains recovered from human blood (n = 349, Appendix 2 Table 2) were retrieved from GenBank (accessed 2018 Nov 1). These *Enterobacter* genomes were subjected to precise species identification by using the Genome-to-Genome Distance Calculator as described.

### Clonal Relatedness on the Basis of Single-Nucleotide Polymorphisms

We performed single-nucleotide polymorphism (SNP) calling for genome sequences to untangle the clonal relatedness of ST171 strains and to investigate whether the ST171 strains in this study are clonally related to strains recovered elsewhere. All genome sequences of ST171 *Enterobacter* strains (n = 102), regardless of types of the host source (human, nonhuman, or unknown) and source (blood, nonblood, or unknown), were retrieved from GenBank. We used the complete chromosome sequence of ST171 strain 34798 (GenBank accession no. CP012165), which was recovered from a bile sample in the United States in 2011, as our reference for mapping. Genome sequences of the strains were mapped against the reference genome by using Snippy version 4.3.6 (https://github.com/tseemann/snippy) at default settings. The resulting core SNPs (n = 1,918) were concatenated and used to infer a phylogenomic tree by using RAxML version 8.2.12 (*17*) under the general time-reversible plus gamma model with a 1,000-bootstrap test.

### Long-Read Genome Sequencing and Plasmid Analysis for ST171 Strains

We determined plasmid replicons of all ST171 strains in this study by using PlasmidFinder version 2.0 (https://cge.cbs.dtu.dk/services/PlasmidFinder). The first *bla*_NDM-5_–harboring ST171 strain (090011) in this study and the only *bla*_NDM-1_–harboring ST171 strain (045001) were selected for whole-genome sequencing by using the long-read MinION sequencer (Nanopore, https://nanoporetech.com) to obtain complete chromosomal and plasmid sequences. De novo hybrid assembly of short Illumina reads and long MinION reads was performed by using Unicycler version 0.4.3 (*15*) in conservative mode for increased accuracy. Complete circular contigs were then corrected by using Pilon version 1.22 (*18*) with Illumina reads for several rounds until no further improvements were reported. Short reads of the remaining four *bla*_NDM-5_–harboring ST171 strains (090022, 090023, 090055, and 090059) were mapped against the *bla*_NDM-5_-carrying plasmid (designated pNDM5_090011) of strain 090011 by using BWA version 0.7.17 (H. Ling, unpub. data, https://arxiv.org/abs/1303.3997) at default settings.

### Statistical Analysis

Continuous variables were presented as median and interquartile range and were compared by using rank-sum test. We used the Pearson χ^2^ test, Yates correction for continuity, or Fisher exact test to compare disparities between different groups for categorical variables. Pearson χ^2^ test was used when sample size (n) was >40 and theoretical frequency (T) >5, Yates correction for continuity when n>40 and 1<T<5, and Fisher exact test when n<40 or T<1. We used SPSS Statistics 21.0 (IBM Inc., https://www.ibm.com) to perform statistical analyses. All p values were 2-tailed, and p<0.05 was considered statistically significant. We used PASS version 11.0 (NCSS, https://www.ncss.com) to calculate statistical power after using the Wilcoxon test to conduct nonparametric adjustment.

Draft genome sequences of the strains in this study were deposited into GenBank (accession numbers in Appendix 2 Table 1). The complete genome sequence of strain 090011 was deposited into GenBank under accession nos. CP036310–2. and the sequence for strain 045001 was deposited under accession nos. CP043382–5.

## Results

### Precise Species Identification of *Enterobacter* Strains

A total of 48 nonduplicated *Enterobacter* strains were recovered from blood during our 2.5-year study period and were collected for study (Table 1; Appendix 2 Table 1). Whole-genome sequencing results, isDDH, and ANI values of these strains are summarized in Appendix 2 Table 1. The 48 strains were identified as *E. cloacae* (n = 42), *E. asburiae* (n = 3), and *E. kobei* (n = 3) by Vitek II. However, precise species identification on the basis of isDDH revealed that the most common species was actually *E. xiangfangensis* (n = 21) (Table 2); the next most common was an *Enterobacter* sp. (n = 8) that has no assigned species name but was previously known as *Enterobacter* cluster III, as defined by Hoffman et al. (*6*). This species is most closely related to *E. xiangfangensis* with a 66.6% isDDH value and is temporarily assigned taxon 1 here (Appendix 2 Table 2). The remaining strains were assigned to 10 *Enterobacter* species: *E. bugandensis* (n = 4), *E. cloacae* (n = 3), *E. asburiae* (n = 2), *E. hormaechei* (n = 2), *E. huaxiensis* (n = 2), *E. roggenkampii* (n = 2), *E. chuandaensis* (n = 1), *E. ludwigii* (n = 1), *E. sichuanensis* (n = 1), and an unnamed *Enterobacter* sp. (n = 1) (Table 2). The unnamed species is most closely related to *E. roggenkampii* with a 65.4% isDDH value (Appendix 1 Table 2) and is temporarily assigned taxon 2 here.

**Table 1:** *Enterobacter* strains in genomic study of *Enterobacter* bloodstream infection, China*

Strain	Date	Species, by isDDH	ST†	Carbapenemase	BSI types					Hospitalization, d		Death
					P	S	CLA	HA		Before BSI	After BSI	
090034	201706	*Enterobacter asburiae*	N12		+					0	27	
090058	201805	*E. asburiae*	879		+		+	+		22	18	
090031	201711	*E. bugandensis*	N10		+					1	0	+
090283	201610	*E. bugandensis*	N16			+		+		7	6	
090210	201607	*E. bugandensis*	499			+				1	16	
090029	201709	*E. bugandensis*	718	NDM-5	+			+		71	16	+
090028	201708	*E. chuandaensis*	N9			+				1	11	
090005	201706	*E. cloacae*	1	NDM-1	+		+	+		29	11	
090016	201703	*E. cloacae*	519			+		+		17	24	
090014	201712	*E. cloacae*	922			+		+		0	10	
090027	201704	*E. hormaechei*	528			+		+		24	38	
090003	201705	*E. hormaechei*	696		+			+		0	15	
045002	201609	*E. huaxiensis*	N1		+					0	7	
090008	201709	*E. huaxiensis*	N1		+			+		3	23	
090017	201702	*E. ludwigii*	12			+		+		6	8	
045158	201607	*E. roggenkampii*	N2		+			+		3	7	
090037	201608	*E. roggenkampii*	984		+		+	+		14	8	
090032	201712	*E. sichuanensis*	N11		+		+	+		6	16	+
090004	201706	*E. xiangfangensis*	N3		+			+		6	13	
090006	201707	*E. xiangfangensis*	N4		+			+		12	8	
090007	201707	*E. xiangfangensis*	N5		+			+		24	19	
090012	201711	*E. xiangfangensis*	N6			+				0	3	
090018	201705	*E. xiangfangensis*	N7		+		+	+		12	14	+
090020	201712	*E. xiangfangensis*	N8		+		+	+		4	60	
090057	201804	*E. xiangfangensis*	N15	NDM-1	+		+	+		36	43	
090015	201712	*E. xiangfangensis*	50		+					0	2	
090026	201704	*E. xiangfangensis*	50		+			+		11	52	
090035	201712	*E. xiangfangensis*	127		+			+		13	20	
045001	201801	*E. xiangfangensis*	171	NDM-1	+		+	+		8	33	
090011	201710	*E. xiangfangensis*	171	NDM-5	+			+		7	14	+
090022	201802	*E. xiangfangensis*	171	NDM-5	+			+		40	20	
090023	201802	*E. xiangfangensis*	171	NDM-5	+		+	+		26	19	
090055	201806	*E. xiangfangensis*	171	NDM-5		+		+		14	14	
090059	201805	*E. xiangfangensis*	171	NDM-5	+		+	+		17	31	
090043	201612	*E. xiangfangensis*	337			+		+		11	4	+
090013	201711	*E. xiangfangensis*	418	NDM-5	+		+	+		28	10	+
090038	201609	*E. xiangfangensis*	418		+			+		3	33	
090060	201806	*E. xiangfangensis*	550		+		+	+		20	8	+
090042	201611	*E. xiangfangensis*	828		+			+		5	3	
090036	201605	Taxon 1	N13		+		+	+		13	22	
090019	201706	Taxon 1	78		+			+		16	62	
090030	201709	Taxon 1	78		+		+	+		0	15	
090039	201610	Taxon 1	78			+		+		12	8	+
090021	201801	Taxon 1	97		+		+	+		5	28	
090009	201709	Taxon 1	104			+		+		5	66	
090033	201704	Taxon 1	316		+			+		10	3	
090056	201803	Taxon 1	568		+			+		11	7	
090040	201611	Taxon 2	N14		+		+	+		208	370	

*BSI, bloodstream infection; CLA, central line–associated; HA, healthcare associated; isDDH, in silico DNA–DNA hybridization; P, primary; S, secondary; ST, sequence type. †There are 16 new sequence types, which are temporarily assigned N1–N16 (Appendix 1 Table 3).

**Table 2:** Proportion of *Enterobacter* species recovered from blood in genomic study of *Enterobacter* bloodstream infection, China

Species*	No. (%)	
	Strains from blood in this study	Strains from blood in GenBank
*Enterobacter xiangfangensis*	21 (43.8)	221 (63.3)
Taxon 1	8 (16.7)	69 (19.8)
*E. bugandensis*	4 (8.3)	7 (2.0)
*E. cloacae*	3 (6.3)	4 (1.1)
*E. asburiae*	2 (4.2)	14 (4.0)
*E. roggenkampii*	2 (4.2)	7 (2.0)
*E. hormaechei*	2 (4.2)	0
*E. huaxiensis*	2 (4.2)	0
*E. ludwigii*	1 (2.1)	6 (1.7)
*E. sichuanensis*	1 (2.1)	1 (0.3)
*E. chuandaensis*	1 (2.1)	0
Taxon 2	1 (2.1)	2 (0.6)
*E. kobei*	0	10 (2.9)
Taxon 3	0	2 (0.6)
Taxon 4	0	2 (0.6)
Taxon 5	0	1 (0.3)
Taxon 6	0	1 (0.3)
*E. chengduensis*	0	1 (0.3)
*E. mori*	0	1 (0.3)
Total	48 (100.0)	349 (100.0)

*Taxons 1–6 represent 6 *Enterobacter* spp. without assigned names. Their most closely related *Enterobacter* spp. are listed in Appendix 1 Table 2 and shown in Appendix 1 Figure 1).

Of 349 *Enterobacter* strains in GenBank that were recovered from human blood, most (221, 63.3%) are also *E. xiangfangensis*; taxon 1 is the second most common species with 69 strains (19.8%) (Table 2). The remaining 59 strains (16.9%) belong to 14 species: *E. asburiae* (n = 14 [4.0%]), *E. kobei* (n = 10 [2.9%]), *E. bugandensis* (n = 7 [2.0%]), *E. roggenkampii* (n = 7 [2.0%]), *E. ludwigii* (n = 6 [1.7%]), *E. cloacae* (n = 4 [1.1%]), taxon 1 (n = 2), *E. chengduensis* (n = 1), *E. mori* (n = 1), *E. sichuanensis* (n = 1), and 4 species without assigned species names (n = 1 or 2 for each species, 6 in total; Table 2). The 4 unnamed species were assigned taxon 3–6 (Table 2); the closest species of taxon 3–6 are listed in Appendix 1 Table 2 and are also shown in a phylogenomic tree in Appendix 1 Figure 1. *E. xiangfangensis* and taxon 1 are closely related as shown by their phylogenetic position in the phylogenomic tree of *Enterobacter* spp. and by their common 66.6% isDDH value (close to the 70% cutoff to define a species). We therefore combined the 2 species in the following analysis.

### BSI Types and Characteristics

Most of the 48 BSI cases (n = 36, 75%) were primary BSIs, including 16 cases of CLABSI. BSIs in the remaining 12 cases were secondary; original sources were intraabdominal infection (n = 5), cholangitis (n = 3), urinary tract infection (n = 2), wound infection (n = 1), and gastrointestinal tract infection (n = 1). Most (n = 41, 85.4%) BSIs caused by *Enterobacter* spp. were healthcare-associated infections. *E. xiangfangensis* and taxon 1 were more likely to cause primary BSI (82.8% vs. 63.2%), CLABSI (27.9% vs. 17.2%), and healthcare-associated BSI (93.1% vs. 73.7%) than were other *Enterobacter* spp. However, the differences were not statistically significant (Table 3).

**Table 3:** Patient demographics, types of bloodstream infection, and outcomes in patients with bloodstream infection caused by *E. xiangfangensis* plus taxon 1 and other *Enterobacter* species, China*

Characteristic	*E. xiangfangensis* and taxon 1, n = 29	Other species, n = 19	χ^2^	p value	Power†
Age, y, median (IQR)	15 (8–32)	52 (16–71)	–	0.958	0.085
Male sex	19 (65.5)	14 (73.7)	0.356	0.551	0.084
MDR	**16 (55.2)**	**2 (10.6)**	**9.763**	**0.002**	–
Primary BSI	24 (82.8)	12 (63.2)	1.423	0.233	0.317
CLABSI	11 (27.9)	5 (17.2)	0.697	0.404	0.115
HA BSI	27 (93.1)	14 (73.7)	2.091	0.148	0.430
Deaths	6 (20.7)	3 (15.8)	0.176	0.675	0.053
Total time hospitalized, d, median (IQR)‡	33 (19–47)	20 (2–40)	–	0.098	0.265
Time hospitalized before BSI onset, d, median (IQR)‡	11 (5–7)	5 (1–18)	–	0.154	0.070
Time hospitalized after BSI onset, d, median (IQR)	15 (8–32)	13 (8–19)	–	0.357	0.384

*Values are no. (%) except as indicated. Bold indicates significance. BSI, bloodstream infection; CLABSI, central line–associated bloodstream infection; HA BSI, healthcare-associated bloodstream infection; IQR, interquartile range; MDR, multidrug resistance; –, not calculated.  †Statistical power was calculated for parameters without statistical significance (p>0.05).  ‡One patient belonging to the other species group had an unusually lengthy hospitalization (578 d) because of a medical dispute and was therefore removed from the analysis of hospitalization time.

Two patients who had *Enterobacter* BSIs (1 *E. xiangfangensis* and 1 taxon 1) died in the hospital. In addition, 7 patients with *Enterobacter* BSIs (4 *E. xiangfangensis*, 2 *E. bugandensis,* and 1 *E. sichuanensis*) did not respond to treatment and were discharged in critical condition. These 7 case-patients were categorized as patients in whom death was predicted. The death rate for *Enterobacter* BSI was 18.8% (9/48); the death rate (20.7% [6/29]) of BSI caused by *E. xiangfangensis* or taxon 1 was not statistically different (15.8% [3/19] p>0.05) (Table 3) from that of BSI caused by other *Enterobacter* spp. Of note, 2 of the 4 patients with *E. bugandensis*–caused BSI had poor outcomes (predicted death) (Table 1). BSIs caused by *E. xiangfangensis* or taxon 1 were more common in younger patients and resulted in lengthier overall hospitalizations (median 33 vs. 19.5 d; p>0.05) (Table 3) than BSIs caused by other *Enterobacter* spp. This difference was largely because of the prolonged length of stay (median 11 d vs. 4.5 d; p>0.05) before the episode of BSI.

### Antimicrobial Susceptibility and Antimicrobial Resistance Genes

The antimicrobial susceptibility and antimicrobial resistance gene repertoire of the 48 *Enterobacter* strains are shown in Appendix 2 Table 1. *E. xiangfangensis* and taxon 1 had substantially higher rates of resistance to aztreonam (48.3% vs. 10.5%), cefepime (41.4% vs. 10.5%), ceftriaxone (58.6% vs. 15.8%), piperacillin/tazobactam (41.4% vs. 10.5%), and tobramycin (44.8% vs. 5.3%) (Table 4) and were substantially more likely to be multidrug resistant (55.2% vs. 10.6%) (Table 3). There were 10 carbapenem-resistant strains (8 *E. xiangfangensis*, 1 *E. bugandensis,* and 1 *E. cloacae*), all of which carried a *bla*_NDM_ gene (*bla*_NDM-5_, n = 7; *bla*_NDM-1_, n = 3) (Table 1; Appendix 2 Table 1), and they belonged to 5 STs (ST171 [n = 6], ST718 [n = 1], ST1 [n = 1], ST418 [n = 1], and a new ST [n = 1]). No carbapenemase genes were identified in carbapenem-susceptible strains.

**Table 4:** Antimicrobial resistance rates in *E. xiangfangensis* plus taxon 1 and other *Enterobacter* spp. in genomic study of *Enterobacter* bloodstream infection, China*

Antimicrobial agent	No. (%)		χ^2^	p value	Power†
	*E. xiangfangensis* + taxon 1, n = 29	Other species, n = 19			
Amikacin	2 (6.9)	0	–	0.512	ND
Gentamicin	9 (31.0)	1 (5.3)	3.192	0.074	0.600
Tobramycin	**13 (44.8)**	**1 (5.3)**	**8.698**	**0.003**	–
Aztreonam	**14 (48.3)**	**2 (10.5)**	**7.361**	**0.007**	**0.829**
Cefepime	**12 (41.4)**	**2 (10.5)**	**5.289**	**0.021**	–
Ceftriaxone	**17 (58.6)**	**3 (15.8)**	**8.664**	**0.003**	–
Imipenem	8 (27.6)	2 (10.5)	1.123	0.289	0.258
Piperacillin/tazobactam	**12 (41.4)**	**2 (10.5)**	**5.289**	**0.021**	–
Ciprofloxacin	**11 (37.9)**	**0**	**7.326**	**0.007**	ND
Levofloxacin	**11 (37.9)**	**0**	**7.326**	**0.007**	ND
Colistin	10 (34.5)	6 (31.6)	0.044	0.835	0.052
Tigecycline	5 (17.2)	0	2.043	0.153	ND
Trimethoprim/sulfamethoxazole	**12 (41.4)**	**0**	**8.392**	**0.004**	ND

*Bold indicates significance. ND, not determined; –, not calculated.  †Statistical power was calculated for parameters without statistical significance (p>0.05) but could not be calculated for any parameters being 0.

### Sequence Types and Clonal Relatedness

The 48 *Enterobacter* strains belonged to 37 STs (Table 1; Appendix 1 Table 3) but only ST171 (*E. xiangfangensis*) and ST78 (taxon 1) contained >3 strains (6 for ST171 and 3 for ST78). We performed analysis of clonal relatedness based on SNPs for ST78 and ST171 strains. In the 3 ST78 strains, there were 306 to 1,052 SNPs difference, suggesting no recent shared origins (Appendix 1 Table 4). The 6 ST171 strains were all resistant to carbapenems and carried *bla*_NDM-5_ (n = 5) or *bla*_NDM-1_ (n = 1). Among the 6 ST171 strains, 4 (all carrying *bla*_NDM-5_) had 0–2 SNPs difference (Table 5) and were recovered from patients in the same ward (cardiac surgery). One patient infected with strain 090011 died but the remaining 3 patients recovered.

**Table 5:** Single-nucleotide polymorphisms between the 6 ST171 strains in genomic study of *Enterobacter* bloodstream infection, China, and strain EC_849 from South Africa and strain CCBH10892 from Brazil*

Strain	090011	090022	090023	090059	045001	090055	EC_849	CCBH10892
090011	–	1	0	1	81	81	106	102
090022	1	–	1	2	82	82	107	103
090023	0	1	–	1	81	81	106	102
090059	1	2	1	–	82	82	107	103
045001	81	82	81	82	–	38	103	99
090055	81	82	81	82	38	–	103	99
EC_849	106	107	106	107	103	103	–	98
CCBH10892	102	103	102	103	99	99	98	–
*–, not calculated.								

The remaining 2 ST171 strains, 045001 (carrying *bla*_NDM-1_) and 090055 (carrying *bla*_NDM-5_), were 81–82 SNPs different from the 4 previously mentioned strains and were 38 SNPs different from each other. These 2 strains were recovered from patients in 2 different wards (medical and respiratory intensive care units). The 6 ST171 strains isolated in our study formed a phylogenetic cluster with strain CCBH10892, which was isolated from a rectal swab sample in Brazil in 2012 (GenBank accession no. JSBO00000000), and strain EC_849, which was isolated from a sputum sample in South Africa in 2012 (GenBank accession no. LRIZ00000000) (Appendix 1 Figure 2). The cluster contained 98 to 107 SNPs difference (Table 5; Appendix 1 Figure 2). Of note, both CCBH10892 and EC_849 carried *bla*_NDM-1_. By contrast, the 6 strains were >300 SNPs different from other ST171 strains with genome sequences deposited in GenBank (Appendix 2 Table 3).

### Plasmid Analysis of ST171 Strains

The complete genome sequences of *bla*_NDM-5_–harboring strain 090011 and the *bla*_NDM-1_–harboring strain 045001 were obtained. Strain 090011 had a 4.64-Mb circular chromosome and 2 plasmids (a 102.5-kb plasmid containing IncFIA, IncFIB, and IncR replicons and a 46.1-kb plasmid containing an IncX3 replicon) (Appendix 1 Table 5). The *bla*_NDM-5_ gene in strain 090011 was carried on the 46.1-kb IncX3 plasmid, designated pNDM5_090011. The short reads of the remaining 4 *bla*_NDM-5_–harboring strains were then mapped against pNDM5_0900117. The 4 strains had contigs showing 100% coverage and 100% identity with pNDM5_090011, suggesting a common plasmid in all 5 *bla*_NDM-5_–harboring ST171 strains in our study. Strain 045001 had a 4.70-Mb circular chromosome and 3 plasmids (an 85.7-kb IncFII plasmid, a 78.2-kb plasmid, and a 2.5-kb plasmid) (Appendix 1 Table 5). The replicon type of the latter 2 plasmids could not be determined by the current replicon-typing scheme. The *bla*_NDM-1_ gene in strain 045001was carried on the 85.7-kb IncFII plasmid.

## Discussion

Although genome sequences deposited in GenBank might be biased in sampling, they can provide complementary information on the species distribution of *Enterobacter* in cases of BSI. Examination of our set of strains and those available in GenBank demonstrates that a variety of *Enterobacter* spp. can cause BSI, but most BSI-causing *Enterobacter* strains belong to either *E. xiangfangensis* or, less commonly, taxon 1. *E. xiangfangensis* and taxon 1 are closely related, with a 66.6% isDDH value (near the 70% cutoff to define a species). Why the 2 species account for most *Enterobacter* BSIs, however, remains unknown. Although the colonization of the human gastrointestinal tract by *Enterobacter* has not been investigated to the level of precise species identification, *E. xiangfangensis* and taxon 1 could be the most common *Enterobacter* species colonizing there, which warrants further study. Alternatively, *E. xiangfangensis* and taxon 1 could be more pathogenic than other *Enterobacter* spp., which also requires further study.

BSIs caused by *E. xiangfangensis* and taxon 1 were more likely to occur in younger patients and result in longer overall hospital stays, although the differences in length of hospitalization between the 2 groups were not statistically significant (p>0.05). This finding might be because of the relatively small sample size (power <0.8) (Table 3). Resistance rates to certain antimicrobial agents, including aztreonam, cefepime, ceftriaxone, piperacillin/tazobactam, and tobramycin and prevalence of multidrug resistance in *E. xiangfangensis* and taxon 1 were substantially higher than in other *Enterobacter* spp. This difference suggests that the identification of *Enterobacter* strains to precise species level also has implications in options of antimicrobial treatment. Although BSI caused by *E. xiangfangensis* and taxon 1 was not associated with higher death rates, 2 of the 4 patients with *E. bugandensis* had poor outcomes (predicted death). *E. bugandensis* has been reported to be a highly pathogenic species associated with life-threatening BSIs and sepsis (*19*). The virulence of this species warrants further study.

As previously noted, 4 *bla*_NDM-5_–harboring ST171 strains had 0–2 SNPs difference (Table 5) and were taken from patients in the same ward (cardiac surgery). The first patient with BSI caused by a strain belonging to the clone (090011) had *Enterobacter* BSI before being transferred to West China Hospital from another facility. Although the particular *Enterobacter* strain from the first hospital was not available for analysis, it is very likely that 090011 was introduced to West China Hospital by transfer of this patient. The next 3 cases were acquired in West China Hospital, highlighting both interhospital and intrahospital transmission of a common strain. BSI in 2 of the 3 cases was CLABSI. These findings suggest that the 4 strains belong to a common clone that caused a cluster of BSI cases. In the hospital ward, central lines were commonly used for drawing blood but were not properly decontaminated after each use; in addition, healthcare workers were observed by anonymous interns to have low compliance (24.8%) with hand hygiene standards established by the World Health Organization. There have been no further *Enterobacter* BSIs after restricting access to central lines and promoting hand hygiene among healthcare workers, which resulted in the compliance rate increasing to 45.5%.

The remaining 2 ST171 strains, 045001 (harboring *bla*_NDM-1_) and 090055 (harboring *bla*_NDM-5_), belong to 2 clones (38 SNPs between each other) which differed from the aforementioned clone by 81–82 SNPs. The relatively low number of SNPs among the 3 ST171 clones also suggests recent divergence within the lineage. In addition, the 6 ST171 strains in this study were clustered together with 2 *bla*_NDM-1_–harboring strains, strain CCBH10892 isolated from Brazil in 2012, and strain EC_849 from South Africa in 2012, with 98 to 107 SNPs, but had >300 SNPs with other ST171 strains that had genome sequences deposited in GenBank. This finding suggests that the 6 strains identified in this study, CCBH10892, and EC_849 represent a subclade of ST171, which carries *bla*_NDM_, has an international distribution, and might have emerged within the past 10 years. In addition, although 090055 and the other 4 *bla*_NDM-5_–harboring strains (090011, 090022, 090023, and 090059) belonged to 2 different clones, they had the same IncX3 plasmid carrying *bla*_NDM-5_, which suggests that the spread of *bla*_NDM-5_ in the hospital was both clonal (vertical) and plasmidborne (horizontal).

The association of ST171 *E. xiangfangensis* with outbreaks is not rare; repeated reports from different geographic locations have demonstrated the same association (*20*–*24*). This association suggests that ST171 is a lineage of *Enterobacter*, which might be well adapted to causing infections in healthcare settings. Previous reports have demonstrated that ST171 *E. xiangfangensis* is a high-risk clone mediating the spread of carbapenem resistance (*21*,*23*). Its emergence was initially documented in 2015 and 2016 by 2 studies in the United States (*24*,*25*). Subsequent studies have revealed the international distribution of ST171 (*21*–*23*). Almost all carbapenem-resistant ST171 *E. xiangfangensis* strains have *bla*_KPC_ (*21*), and only a small number of strains carry *bla*_NDM_ instead (*21*,*26*). In this study, we identified in-hospital transmission of carbapenem-resistant ST171 strains, which carried *bla*_NDM-5_ rather than *bla*_KPC_ as seen in previous studies (*21*,*23*). The ability to acquire different carbapenemase genes and its adaptability to healthcare settings might be major drivers in the emergence of ST171, which warrants further study.

Our investigation demonstrates the value of whole-genome sequencing for precise species identification. However, this study has several limitations. First, because it is a single site study, the application of our findings could be limited. However, we analyzed genomes available in GenBank to provide the most comprehensive information possible. Second, the relatively small sample size in this study might not have adequate power to examine statistical significance in BSI type and patient outcomes. However, this study provided useful information on the clinical importance of *E. xiangfangensis* and its closely related taxon 1. Larger-scale studies are warranted.

In conclusion, most *Enterobacter* strains recovered from human blood in China were not *E. cloacae* but *E. xiangfangensis*. Most *Enterobacter* BSI cases in our study were healthcare-associated and primary infections. *E. xiangfangensis* ST171 is a major lineage of carbapenem-resistant *Enterobacter*, has an intercontinental distribution, is usually healthcare associated, and carries *bla*_NDM_ rather than *bla*_KPC_ in China. Precise species identification of *Enterobacter* has clinical importance in antimicrobial therapy and infection control.

**Addendum:** Since submission and acceptance of this manuscript, the taxonomy of *Enterobacter* has been substantially updated. The updated *Enterobacter* taxonomy is available at https://doi.org/10.1128/mSystems.00527-20. The update of new taxa identified in this study is shown in Appendix 3 Table.

Appendix 1 Additional information about precise species identification by whole-genome sequencing of *Enterobacter* bloodstream infection, China.

Appendix 2 Additional information about precise species identification by whole-genome sequencing of *Enterobacter* bloodstream infection, China.

Appendix 3 Additional information about precise species identification by whole-genome sequencing of *Enterobacter* bloodstream infection, China.
