# Supplementary material for: Precise Species Identification by Whole-Genome Sequencing of Enterobacter Bloodstream Infection, China
Source: Emerg Infect Dis. 2021 Jan;27(1):161–9. doi: 10.3201/eid2701.190154 (PMC7774573; doi:10.3201/eid2701.190154)
Supplement: Appendix 1 — Additional information about precise species identification by whole-genome sequencing of Enterobacter bloodstream infection, China. [file 19-0154-Techapp-s1.pdf]

Article DOI: https://doi.org/10.3201/eid2701.190154

Precise Species Identification by Whole-Genome Sequencing of *Enterobacter* Bloodstream Infection, China

Appendix 1

Appendix 1 Table 1. Classification and nomenclature of the genus *Enterobacter* as of December 2018*

Species	Hoffman cluster	Reference	Type strain	GenBank accession no. or current species name
Species name for validation (n = 14)				
Enterobacter asburiae	I	(*1*)	JCM 6051	CP011863
E. cancerogenus	-	(*2*)	ATCC 35316	ERR1854846
E. chuandaensis	-	(*3*)	090028[T]	QZCS00000000
E. cloacae	XI		ATCC 13047	ERR1854846
E. bugandensis	IX	(*4*)	EB-247	FYBI00000000
E. hormaechei	VII	(*5*)	ATCC 49162	MKEQ00000000
E. huaxiensis	-	(*3*)	090008[T]	QZCT00000000
E. kobei	II	(*6*)	ATCC BAA-260	CP017181
E. ludwigii	V	(*7*)	EN-119	CP017279
E. mori	-	(*8*)	LMG 25706	AEXB00000000
E. soli	-	(*9*)	ATCC BAA-2102	LXES00000000
E. tabaci	-	(*10*)	YIM Hb-3	N/A
E. xiangfangensis†	VI	(*11*)	LMG 27195	CP017183
E. sichuanensis	-	(*12*)	WCHECl1597	POVL00000000
Species in doubt (n = 2)				
E. muelleri‡	-	(*13*)	JM-458	FXLQ00000000
E. siamensis§	-	(*14*)	C2361	N/A
Species name awaiting validation (n = 3)				
E. timonensis	-	(*15*)	mt20	FCOP00000000
E. chengduensis	-	(*16*)	WCHECl-C4	MTSO00000000
E. roggenkampii	IV	(*17*)	DSM16690	CP017184
Species listed in LPSN but moved out of *E.* (n = 20)				

Species	Hoffman cluster	Reference	Type strain	GenBank accession no. or current species name
E. aerogenes		(*18*)	ATCC 13048	*Klebsiella aerogenes*
E. agglomerans		(*19*)	ATCC 27155	*Pantoea agglomerans*
E. amnigenus		(*20*)	ATCC 33072	*Lelliottia amnigena*
E. arachidis		(*20*)	KCTC 22375	*Kosakonia arachidis*
E. cowanii		(*20*)	CCUG 45998	*Kosakonia cowanii*
E. gergoviae		(*20*)	ATCC 33028	*Pluralibacter gergoviae*
E. helveticus		(*20*)	JCM 16470	*Cronobacter helveticus*
E. intermedius		(*21*)	ATCC 33110	*Kluyvera intermedia*
E. massiliensis		(*22*)	JC163	*Metakosakonia massiliensis*
E. nimipressuralis	X	(*20*)	CIP 104980	*Lelliottia nimipressuralis*
E. oryzae		(*20*)	LMG 24251	*Kosakonia oryzae*
E. oryzendophyticus		(*23*)	LMG 26432	*Kosakonia oryzendophytica*
E. oryziphilus		(*23*)	LMG 26429	*Kosakonia oryziphila*
E. pulveris		(*20*)	DSM 19144	*Cronobacter pulveris*
E. pyrinus		(*20*)	ATCC 49851	*Pluralibacter pyrinus*
E. radicincitans		(*20*)	CIP 108468	*Kosakonia radicincitans*
E. sacchari		(*11*)	CGMCC 1.12102	*Kosakonia sacchari*
E. sakazakii		(*24*)	ATCC 29544	*Cronobacter sakazakii*
E. taylorae		(*25*)	ATCC 35317	*Enterobacter cancerogenus*
E. turicensis		(*20*)	DSM 18397	*Cronobacter zurichensis*

*LPSN, The list of Prokaryotic Names with Standing in Nomenclature.

†The species status of *E. xiangfangensis* has been doubted previously and it has been proposed as a subspecies of *E. hormaechei* rather than a valid species (*17,26*). However, its type strain has only 94.48% ANI and 60.0% isDDH with *E. hormaechei* type strain ATCC 49162^{T} (GenBank accession no. MKEQ00000000). Therefore, *E. xiangfangensis* and *E. hormaechei* are clearly 2 different species.

‡*E. mueller* is a later synonym of *E. asburiae*.

§It has been proposed to reject *E. siamensis* because the 16S rRNA sequence of its type strain available in collections does not match its record in GenBank (*27*).

Appendix 1 Table 2. The 6 unnamed *Enterobacter* spp. identified in genomic study of *Enterobacter* bloodstream infection, China*

Species assignation	Representative strain	Genome accession no.	Closest species	isDDH (%)†
Taxon 1	DSM 14563‡	CP017186	*E. xiangfangensis*	66.6
Taxon 2	e362	FKDT00000000	*E. roggenkampii*	65.4
Taxon 3	e773	FKGE00000000	*E. asburiae*	65.3
Taxon 4	e2032	FKBK00000000	*E. asburiae*	52.0
Taxon 5	e483	FKEG01000000	*E. asburiae*	49.6
Taxon 6	153C2	QMCQ01000000	*E. xiangfangensis*	52.8

*isDDH, in silico DNA–DNA hybridization.

†isDDH values between the representative strain and the type strain of closest species.

‡Strain DSM 14563 has been proposed as the type strain of E. hormaechei subspecies Hoffmannii. However, the strain has only 94.13% ANI and 58.0% isDDH with E. hormaechei type strain ATCC 49162T (GenBank accession no. MKEQ00000000). It is clear that the E. hormaechei subspecies hoffmannii is actually not a subspecies of E. hormaechei but rather represents a new, unnamed Enterobacter species. In this study, we temporarily designated the species taxon 1 for simplicity.

Appendix 1 Table 3. Profiles of sequence types in genomic study of *Enterobacter* bloodstream infection, China*

ST	Species	*dnaA*	*fusA*	*gyrB*	*leuS*	*pyrG*	*rplB*	*rpoB*	Closest ST† (no. of allele difference)
1	*E. cloacae*	1	1	1	1	1	1	1	
12	*E. ludwigii*	13	2	45	24	52	2	14	
50	*E. xiangfangensis*	4	4	4	6	37	4	25	
78	Taxon 1	8	9	6	9	9	6	8	
97	Taxon 1	59	9	62	9	62	25	6	
104	Taxon 1	59	40	76	9	70	6	6	
127	*E. xiangfangensis*	46	20	74	44	45	24	6	
171	*E. xiangfangensis*	49	21	19	44	45	12	32	
316	Taxon 1	59	88	82	9	67	6	6	
337	*E. xiangfangensis*	67	21	9	129	45	12	32	
418	*E. xiangfangensis*	53	35	154	44	45	4	6	
499	*E. bugandensis*	164	18	183	200	120	8	29	
519	*E. cloacae*	1	107	158	1	168	36	1	
528	*E. hormaechei*	95	56	112	116	104	4	63	
550	*E. xiangfangensis*	179	4	4	6	112	4	6	
568	Taxon 1	189	9	12	9	67	6	6	
696	*E. hormaechei*	225	140	93	268	224	109	141	
718	*E. bugandensis*	140	18	248	31	230	8	29	
828	*E. xiangfangensis*	9	4	14	61	257	4	9	
879	*E. asburiae*	152	15	102	15	101	11	133	

ST	Species	*dnaA*	*fusA*	*gyrB*	*leuS*	*pyrG*	*rplB*	*rpoB*	Closest ST† (no. of allele difference)
922	*E. cloacae*	169	107	61	168	36	77	1	
984	*E. roggenkampii*	65	57	49	94	49	12	47	
N1	*E. huaxiensis*	n1	n1	n1	n1	n1	n1	n1	None
N2	*E. roggenkampii*	191	n2	254	193	49	12	26	613 (3)
N3	*E. xiangfangensis*	n2	20	148	44	45	4	6	886/916/986 (1)
N4	*E. xiangfangensis*	4	4	15	4	11	30	6	111/981 (2)
N5	*E. xiangfangensis*	58	22	14	6	39	4	9	79 (1)
N6	*E. xiangfangensis*	58	41	14	6	69	4	n2	106 (1)
N7	*E. xiangfangensis*	178	4	4	6	92	4	6	542 (1)
N8	*E. xiangfangensis*	4	37	4	6	42	4	6	329 (1)
N9	*E. chuandaensis*	n3	n3	n2	n2	n2	n2	120	573/944 (6)
N10	*E. bugandensis*	309	18	n3	n3	34	8	n3	1084 (3)
N11	*E. sichuanensis*	n4	98	170	n4	n3	68	n4	472/607/738/847 (4)
N12	*E. asburiae*	n5	15	n4	124	n4	11	68	319 (3)
N13	Taxon 1	59	9	n5	n5	79	37	n5	157/419/792 (4)
N14	Taxon 2	151	108	n6	n6	n5	14	93	474 (3)
N15	*E. xiangfangensis*	n6	69	19	44	64	4	32	270 (2)
N16	*E. bugandensis*	140	18	n7	31	230	8	29	718 (1)

*N1 to N16 are new sequence types. New alleles are temporarily assigned n1 to n6. ST, sequence type.

†For new sequence types only.

Appendix 1 Table 4. Single nucleotide polymorphisms between the 3 ST78 strains in genomic study of *Enterobacter* bloodstream infection, China*

Strain	090039	090030	090019
090039	–	1,052	814
090030	1,052	-	306
090019	814	306	–

*The 3 genomes were mapped against the complete chromosome sequence of ST78 strain AR_0050 (GenBank accession no. CP021896) by using Parsnp version 1.2 and alignment was obtained by using Harvest (28).

Appendix 1 Table 5. Complete genome and antimicrobial resistance genes of strain 090011 and strain 045001 in genomic study of *Enterobacter* bloodstream infection, China*

			Genes mediating resistance to							
Strain	Size, bp	Replicon type, Inc	β-lactam	Aminoglycoside	Fluoroquinolone	Fosfomycin	Rifampin	Sulfonamide	Tetracycline	Trimethoprim
090011										
Chromosome	4,639,926	-	*bla*$_{ACT-7}$			*fosA*				
pNDM5_090011	46,161	X3	*bla*$_{NDM-5}$							
pCTXM65_090011	102,543	FIA, FIB, R	*bla*$_{CTX-M-65}$, *bla*$_{TEM-1B}$	*aadA16,* *aac(6')-Ib*-cr	*aac(6')-Ib*-cr		*arr-3*	*sul1*	*tet*(A)	*dfrA27*
045001										
Chromosome	4,698,270	-	*bla*$_{CTX-M-15}$, *bla*$_{OXA-1}$, *bla*$_{ACT-7}$	*aac(3)-IIa,* *aac(6')-Ib*-cr	*aac(6')-Ib*-cr, *qnrB1*	*fosA*				
pNDM1_045001	85,718	FII	*bla*$_{NDM-1}$, *bla*$_{TEM-1}$	*rmtB*					*tet*(A)	*dfrA14*
p1_045001	78,247	ND								
p2_045001	2,496	ND								

*ND, undetermined.

References

1. Brenner DJ, McWhorter AC, Kai A, Steigerwalt AG, Farmer JJ III. *Enterobacter asburiae* sp. nov., a new species found in clinical specimens, and reassignment of *Erwinia dissolvens* and *Erwinia nimipressuralis* to the genus *Enterobacter* as *Enterobacter dissolvens* comb. nov. and *Enterobacter nimipressuralis* comb. nov. J Clin Microbiol. 1986;23:1114–20. PubMed https://doi.org/10.1128/JCM.23.6.1114-1120.1986

2. Dickey RS, Zumoff CH. Emended description of *Enterobacter cancerogenus* comb. nov. (formerly *Erwinia cancerogena*). Int J Syst Bacteriol. 1988;38:371–4. https://doi.org/10.1099/00207713-38-4-371

3. Wu W, Wei L, Feng Y, Kang M, Zong Z. *Enterobacter huaxiensis* sp. nov. and *Enterobacter chuandaensis* sp. nov. recovered from human blood. Int J Syst Evol Microbiol. 2018;69:708–14 In press. PubMed https://doi.org/10.1099/ijsem.0.003207

4. Doijad S, Imirzalioglu C, Yao Y, Pati NB, Falgenhauer L, Hain T, et al. *Enterobacter bugandensis* sp. nov., isolated from neonatal blood. Int J Syst Evol Microbiol. 2016;66:968–74. PubMed https://doi.org/10.1099/ijsem.0.000821

5. O'Hara CM, Steigerwalt AG, Hill BC, Farmer JJ III, Fanning GR, Brenner DJ. *Enterobacter hormaechei*, a new species of the family *Enterobacteriaceae* formerly known as enteric group 75. J Clin Microbiol. 1989;27:2046–9. PubMed https://doi.org/10.1128/JCM.27.9.2046-2049.1989

6. Kosako Y, Tamura K, Sakazaki R, Miki K. *Enterobacter kobei* sp. nov., a new species of the family *Enterobacteriaceae* resembling *Enterobacter cloacae.* Curr Microbiol. 1996;33:261–5. PubMed https://doi.org/10.1007/s002849900110

7. Hoffmann H, Stindl S, Stumpf A, Mehlen A, Monget D, Heesemann J, et al. Description of *Enterobacter ludwigii* sp. nov., a novel *Enterobacter* species of clinical relevance. Syst Appl Microbiol. 2005;28:206–12. PubMed https://doi.org/10.1016/j.syapm.2004.12.009

8. Zhu B, Lou MM, Xie GL, Wang GF, Zhou Q, Wang F, et al. *Enterobacter mori* sp. nov., associated with bacterial wilt on *Morus alba* L. Int J Syst Evol Microbiol. 2011;61:2769–74. PubMed https://doi.org/10.1099/ijs.0.028613-0

9. Manter DK, Hunter WJ, Vivanco JM. *Enterobacter soli* sp. nov.: a lignin-degrading γ-proteobacteria isolated from soil. Curr Microbiol. 2011;62:1044–9. PubMed https://doi.org/10.1007/s00284-010-9809-9

10. Duan YQ, Zhou XK, Di-Yan L, Li QQ, Dang LZ, Zhang YG, et al. *Enterobacter tabaci* sp. nov., a novel member of the genus *Enterobacter* isolated from a tobacco stem. Antonie van Leeuwenhoek. 2015;108:1161–9. PubMed https://doi.org/10.1007/s10482-015-0569-1

11. Gu CT, Li CY, Yang LJ, Huo GC. *Enterobacter xiangfangensis* sp. nov., isolated from Chinese traditional sourdough, and reclassification of *Enterobacter sacchari* Zhu et al. 2013 as *Kosakonia sacchari* comb. nov. Int J Syst Evol Microbiol. 2014;64:2650–6. PubMed https://doi.org/10.1099/ijs.0.064709-0

12. Wu W, Feng Y, Zong Z. *Enterobacter sichuanensis* sp. nov., recovered from human urine. Int J Syst Evol Microbiol. 2018;68:3922–7. PubMed https://doi.org/10.1099/ijsem.0.003089

13. Kämpfer P, McInroy JA, Glaeser SP. *Enterobacter muelleri* sp. nov., isolated from the rhizosphere of *Zea mays*. Int J Syst Evol Microbiol. 2015;65:4093–9. PubMed https://doi.org/10.1099/ijsem.0.000547

14. Khunthongpan S, Bourneow C, H-Kittikun A, Tanasupawat S, Benjakul S, Sumpavapol P. *Enterobacter siamensis* sp. nov., a transglutaminase-producing bacterium isolated from seafood processing wastewater in Thailand. J Gen Appl Microbiol. 2013;59:135–40. PubMed https://doi.org/10.2323/jgam.59.135

15. Tidjani Alou M, Cadoret F, Brah S, Diallo A, Sokhna C, Mehrej V, et al. ‘*Khelaifiella massiliensis*’, ‘*Niameybacter massiliensis*’, ‘*Brachybacterium massiliense*’, ‘*Enterobacter timonensis*’, ‘*Massilibacillus massiliensis*’, new bacterial species and genera isolated from the gut microbiota

of healthy infants. New Microbes New Infect. 2017;19:1–7. PubMed https://doi.org/10.1016/j.nmni.2017.02.002

16. Wu W, Feng Y, Zong Z. Characterization of a strain representing a new *Enterobacter* species, *Enterobacter chengduensis* sp. nov. Antonie van Leeuwenhoek. 2019;112:491–500. PubMed https://doi.org/10.1007/s10482-018-1180-z

17. Granger G. Sutton, Lauren M. Brinkac, Thomas H. Clarke, Fouts DE. *Enterobacter hormaechei* subsp. *hoffmannii* subsp. nov., *Enterobacter hormaechei* subsp. *xiangfangensis* comb. nov., *Enterobacter roggenkampii* sp. nov., and *Enterobacter muelleri* is a later heterotypic synonym of *Enterobacter asburiae* based on computational analysis of sequenced *Enterobacter* genomes. F1000 Res. 2018;7:521. https://doi.org/10.12688/f1000research.14566.1

18. Tindall BJ, Sutton G, Garrity GM. *Enterobacter aerogenes* Hormaeche and Edwards 1960 (approved lists 1980) and *Klebsiella mobilis* Bascomb et al. 1971 (approved lists 1980) share the same nomenclatural type (ATCC 13048) on the approved lists and are homotypic synonyms, with consequences for the name *Klebsiella mobilis* Bascomb et al. 1971 (approved lists 1980). Int J Syst Evol Microbiol. 2017;67:502–4. PubMed https://doi.org/10.1099/ijsem.0.001572

19. Edwards J, Baque E, Tang J. Proposal to acknowledge Beijerinck as the original author of the species *Pantoea agglomerans.* Request for an opinion. Int J Syst Evol Microbiol. 2004;54:2437. PubMed https://doi.org/10.1099/ijs.0.02955-0

20. Brady C, Cleenwerck I, Venter S, Coutinho T, De Vos P. Taxonomic evaluation of the genus *Enterobacter* based on multilocus sequence analysis (MLSA): proposal to reclassify *E. nimipressuralis* and *E. amnigenus* into *Lelliottia* gen. nov. as *Lelliottia nimipressuralis* comb. nov. and *Lelliottia amnigena* comb. nov., respectively, *E. gergoviae* and *E. pyrinus* into *Pluralibacter* gen. nov. as *Pluralibacter gergoviae* comb. nov. and *Pluralibacter pyrinus* comb. nov., respectively, *E. cowanii, E. radicincitans, E. oryzae* and *E. arachidis* into *Kosakonia* gen. nov. as *Kosakonia cowanii* comb. nov., *Kosakonia radicincitans* comb. nov., *Kosakonia oryzae*

comb. nov. and *Kosakonia arachidis* comb. nov., respectively, and *E. turicensis, E. helveticus* and *E. pulveris* into *Cronobacter* as *Cronobacter zurichensis* nom. nov., *Cronobacter helveticus* comb. nov. and *Cronobacter pulveris* comb. nov., respectively, and emended description of the genera *Enterobacter* and *Cronobacter*. Syst Appl Microbiol. 2013;36:309–19. PubMed https://doi.org/10.1016/j.syapm.2013.03.005

21. Pavan ME, Franco RJ, Rodriguez JM, Gadaleta P, Abbott SL, Janda JM, et al. Phylogenetic relationships of the genus *Kluyvera*: transfer of *Enterobacter intermedius* Izard et al. 1980 to the genus *Kluyvera* as *Kluyvera intermedia* comb. nov. and reclassification of *Kluyvera cochleae* as a later synonym of *K. intermedia*. Int J Syst Evol Microbiol. 2005;55:437–42. PubMed https://doi.org/10.1099/ijs.0.63071-0

22. Alnajar S, Gupta RS. Phylogenomics and comparative genomic studies delineate six main clades within the family *Enterobacteriaceae* and support the reclassification of several polyphyletic members of the family. Infect Genet Evol. 2017;54:108–27. PubMed https://doi.org/10.1016/j.meegid.2017.06.024

23. Di Pilato V, Arena F, Tascini C, Cannatelli A, Henrici De Angelis L, Fortunato S, et al. *mcr-1.2*, a new *mcr* variant carried on a transferable plasmid from a colistin-resistant KPC carbapenemase-producing *Klebsiella pneumoniae* strain of sequence type 512. Antimicrob Agents Chemother. 2016;60:5612–5. PubMed https://doi.org/10.1128/AAC.01075-16

24. Iversen C, Lehner A, Mullane N, Bidlas E, Cleenwerck I, Marugg J, et al. The taxonomy of *Enterobacter sakazakii*: proposal of a new genus *Cronobacter* gen. nov. and descriptions of *Cronobacter sakazakii* comb. nov. *Cronobacter sakazakii* subsp. *sakazakii*, comb. nov., *Cronobacter sakazakii* subsp. *malonaticus* subsp. nov., *Cronobacter turicensis* sp. nov., *Cronobacter muytjensii* sp. nov., *Cronobacter dublinensis* sp. nov. and *Cronobacter* genomospecies 1. BMC Evol Biol. 2007;7:64. PubMed https://doi.org/10.1186/1471-2148-7-64

25. Schønheyder HC, Jensen KT, Frederiksen W. Taxonomic notes: synonymy of *Enterobacter cancerogenus* (Urosević 1966) Dickey and Zumoff 1988 and *Enterobacter taylorae* Farmer et al. 1985 and resolution of an ambiguity in the biochemical profile. Int J Syst Bacteriol. 1994;44:586–7. PubMed https://doi.org/10.1099/00207713-44-3-586

26. Chavda KD, Chen L, Fouts DE, Sutton G, Brinkac L, Jenkins SG, et al. Comprehensive genome analysis of carbapenemase-producing *Enterobacter* spp.: new insights into phylogeny, population structure, and resistance mechanisms. MBio. 2016;7:02093–16. PubMed https://doi.org/10.1128/mBio.02093-16

27. Kämpfer P, Doijad S, Chakraborty T, Glaeser SP. The status of the species *Enterobacter siamensis* Khunthongpan et al. 2014. Request for an Opinion. Int J Syst Evol Microbiol. 2016;66:524–5. PubMed https://doi.org/10.1099/ijsem.0.000777

28. Treangen TJ, Ondov BD, Koren S, Phillippy AM. The Harvest suite for rapid core-genome alignment and visualization of thousands of intraspecific microbial genomes. Genome Biol. 2014;15:524. PubMed https://doi.org/10.1186/s13059-014-0524-x

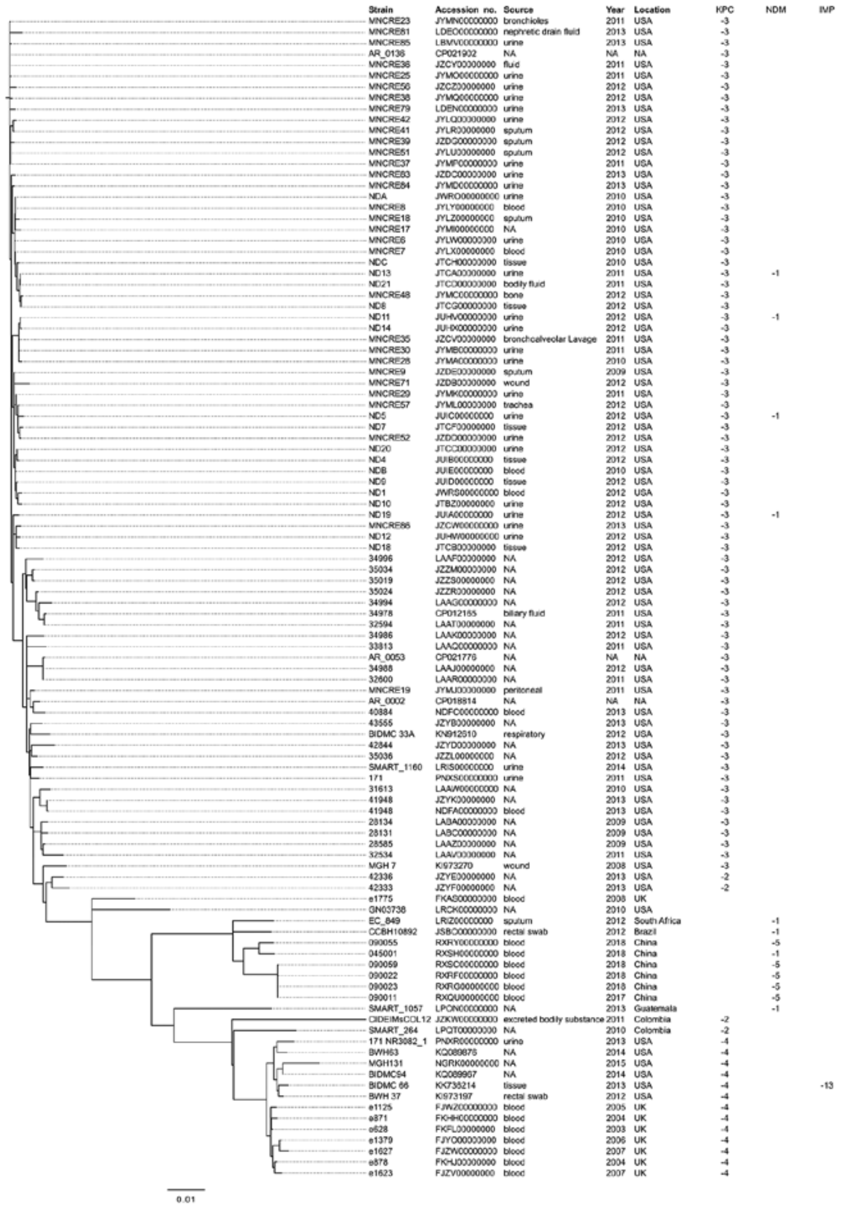

Appendix 1 Figure 1. Phylogenomic tree of *Enterobacter* species. Phylogenomic tree based on the concatenated nucleotide sequence of the core-genes of type strains of species belonging to the genus *Enterobacter* (Appendix 1 Table 1) and representative strains of the 6 new species (Appendix 1 Table 2) as described previously (*12*). The species and strain name are shown and accession numbers are indicated in parentheses. The tree was inferred by using the maximum-likelihood algorithm. Bootstrap values >50% (based on 1,000 resamplings) are indicated by the dotted lines. Scale bar indicates 0.4 nt substitution per site.

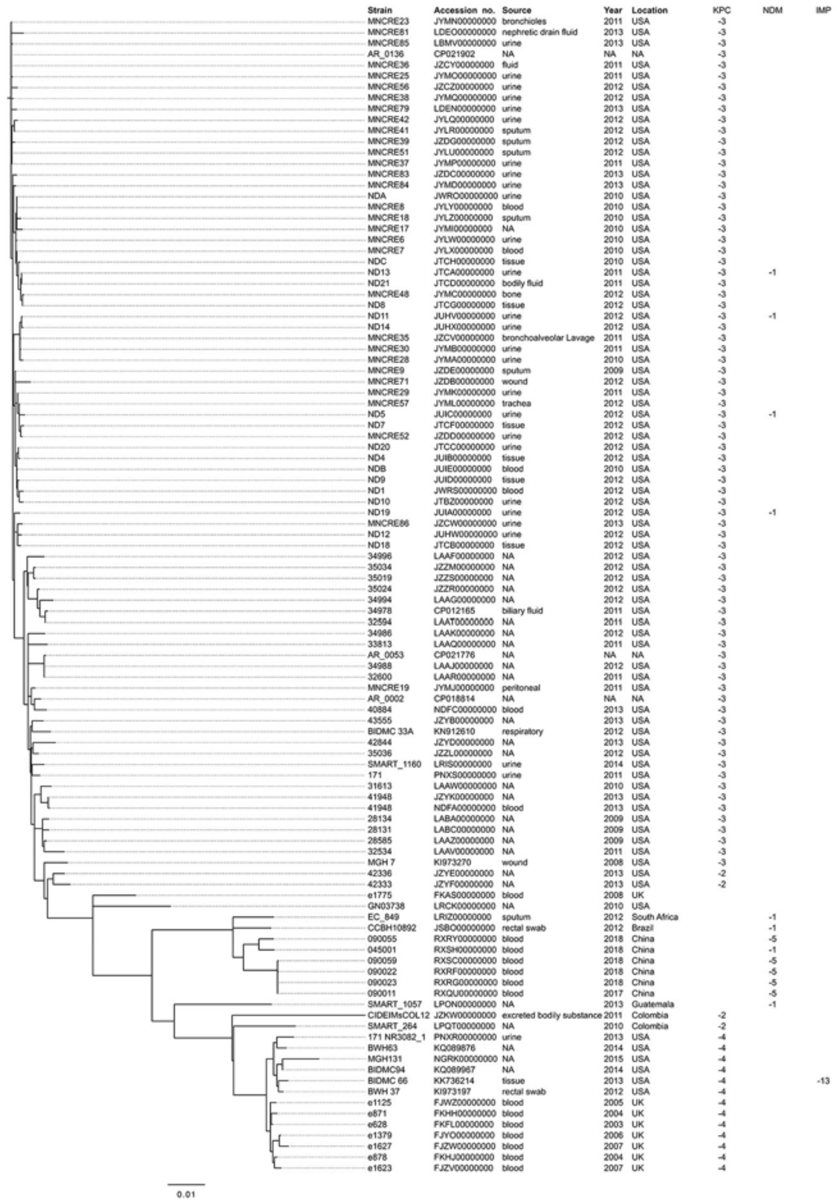

Appendix 1 Figure 2. Phylogenomic tree of ST171 *Enterobacter* strains. Strains in this study are highlighted in red. The strain name, accession no., sample type, year and country of recovery, and carbapenemase genes are shown. Among 108 ST171 strains (102 from GenBank and 6 in this study), 2 strains, e1481 and e1486, had >20,000 single nucleotide polymorphisms compared with other strains (Appendix 2 Table 3), suggesting different origins. These 2 strains were therefore removed from the phylogenomic tree. NA, not available.
