# Supplementary material for: Precise Species Identification by Whole-Genome Sequencing of Enterobacter Bloodstream Infection, China
Source: Emerg Infect Dis. 2021 Jan;27(1):161–9. doi: 10.3201/eid2701.190154 (PMC7774573; doi:10.3201/eid2701.190154)
Supplement: Appendix 3 — Additional information about precise species identification by whole-genome sequencing of Enterobacter bloodstream infection, China. [file 19-0154-Techapp-s3.pdf]

Article DOI: https://doi.org/10.3201/eid2701.190154

Precise Species Identification by Whole-Genome Sequencing of *Enterobacter* Bloodstream Infection, China

Appendix 3

The Update of Taxonomic Assignments of New Taxa Identified in this Study

Since the submission and acceptance of this manuscript, the taxonomy of *Enterobacter* has been substantially updated. The updated *Enterobacter* taxonomy is available at doi: 10.1128/mSystems.00527-20. The update of new taxa identified in this study is shown in the table below.

Appendix 3 Table. The update of taxonomic assignments of new taxons identified in genomic study of *Enterobacter* bloodstream infection

Taxonomic assignment in this study	Assignment in the updated taxonomy at doi: 10.1128/mSystems.00527-20
Taxon 1	*Enterobacter hoffmannii*
Taxon 2	*Enterobacter quasiroggenkampii*
Taxon 3	Taxon14
Taxon 4	Taxon 4
Taxon 5	Taxon 5
Taxon 6	*Enterobacter quasihormaechei*
